# Nutritional, Microbial, and Allergenic Changes during the Fermentation of Cashew ‘Cheese’ Product Using a Quinoa-Based Rejuvelac Starter Culture

**DOI:** 10.3390/nu12030648

**Published:** 2020-02-28

**Authors:** Jennifer M. Chen, Kait F. Al, Laura J. Craven, Shannon Seney, Margaret Coons, Heather McCormick, Gregor Reid, Colleen O’Connor, Jeremy P. Burton

**Affiliations:** 1 Department of Microbiology and Immunology, Schulich School of Medicine, Western University, London, ON N6A 5C1, Canada; jche252@uwo.ca (J.M.C.); kal@uwo.ca (K.F.A.); lcraven2@uwo.ca (L.J.C.); gregor@uwo.ca (G.R.); 2 Lawson Health Research Institute, 268 Grosvenor Street, London, ON N6C 2R5, Canada; shannon.seney@sjhc.london.on.ca; 3 Nuts for Cheese, London, ON N5V 3K4, Canada; info@nutsforcheese.com (M.C.); qa@nutsforcheese.com (H.M.); 4 Department of Surgery, Western University, London, ON N6A 4V2, Canada; 5 Brescia University College, London, ON N6G 1H2, Canada; cgobert@uwo.ca

**Keywords:** fermentation, cashew, allergen, allergy, nuts, cheese

## Abstract

Fermentation has been applied to a multitude of food types for preservation and product enhancing characteristics. Interest in the microbiome and healthy foods makes it important to understand the microbial processes involved in fermentation. This is particularly the case for products such as fermented cashew (*Anacardium occidentale*). We hereby describe the characterisation of cashew samples throughout an entire fermentation production process, starting at the quinoa starter inoculum (rejuvelac). The viable bacterial count was 10^8^ -10^9^ colony forming units/g. The nutritional composition changed marginally with regards to fats, carbohydrates, vitamins, and minerals. The rejuvelac starter culture was predominated by *Pediococcus* and *Weissella* genera. The ‘brie’ and ‘blue’ cashew products became dominated by *Lactococcus*, *Pediococcus*, and *Weissella* genera as the fermentation progressed. Cashew allergenicity was found to significantly decrease with fermentation of all the end-product types. For consumers concerned about allergic reactions to cashew nuts, these results suggested that a safer option is for products to be made by fermentation.

## 1. Introduction

Food fermentation began as a method of preservation, but subsequently developed into a mechanism of altering taste, texture, and nutritional content. The process relies on the metabolic activity of microorganisms to yield by-products that enhance food preservation [1,2]. Fermentation can be undertaken by microorganisms that are either inoculated, already present, or mixed between the two types. [3]. The benefits of convenience and product consistency associated with inoculated fermentation is often matched with a decrease of flavour richness, leading to more bland products [4]. An industrial reality is that the process still mostly depends on spontaneous fermentations to create the enhanced flavour profile that is desired. However, this technique can result in inconsistent products due to a lack of control of the viability of the microorganisms present in the raw ingredients.

Fermentation makes food last longer; one method is through acid production that decreases pH and creates an environment unsuitable for harmful bacteria to survive in. The opportunity exists to create new products using microorganisms that can enrich taste, texture, and smell [4]. The bulk of fermented foods are milk-based, resulting in yoghurt, cheese, and kefir among others. These tend to have a consistent taste, smell, and texture. Common bacteria involved in fermenting milk are *Streptococcus* spp., *Lactobacillus* spp., and *Lactococcus* spp. [5]. A popular dairy product is cheese, which depends on the bacterial fermentation of lactose into lactate and lactic acid [6]. Despite a history of thousands of years, the making of cheese is still evolving, as more efficient and safe methods are introduced [7]. These include dairy-free ‘cheese-like’ products, especially for consumers with allergies or certain dietary restrictions. One example is a fermented cashew ‘cheese-like’ product, where milk is entirely replaced with blended cashews. Although dairy is not used, it is colloquially called “cheese” due to the similarity in taste and texture to spreadable cheese. These cashew products provide an option for people who do not consume dairy but still desire some of the nutrients and flavours associated with cheese. 

A benefit of fermentation is that it can decrease allergenicity or increase tolerance to certain foods [8,9]. The enzymatic activity associated with fermentation breaks down peptides associated with allergens via enzymatic hydrolysis [10,11]. This has been successfully used to decrease the allergenicity of food containing peanut and milk allergens [12,13,14,15,16]. In fact, fermentation is one of the few processing methods that can reduce allergenicity to the point of consumption without allergic reaction [8]. Enzymatic hydrolysis to reduce cashew allergenicity has not yet been demonstrated. Allergy to cashew nuts is common and can elicit more severe reactions than peanuts [17,18]. The aim of this study was to analyze the microbes present throughout the production of cashew ‘cheese’ and assess the nutritional composition in finished products.

## 2. Materials and Methods

### 2.1. Fermented Cashew Cheese-Like Product Production Process

**Figure 1:**
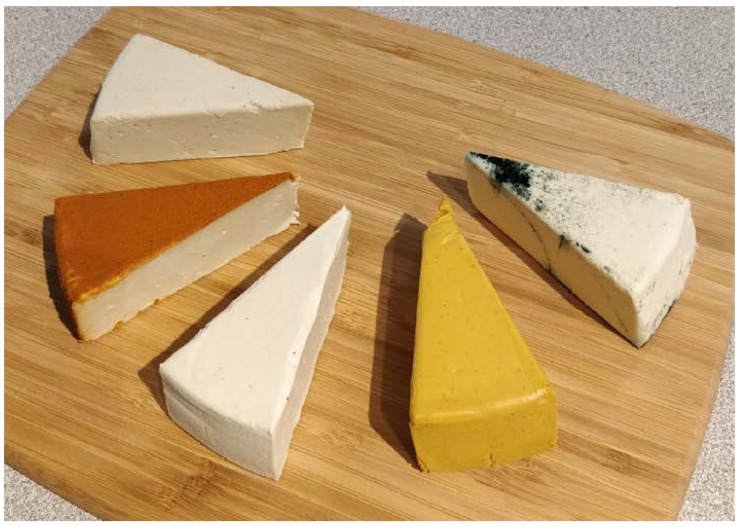
Five flavours of fermented cashew product. From left to right: ‘brie’, red, herb, ‘cheddar’, and ‘blue’ cashew cheese-like product.

**Figure 2:**
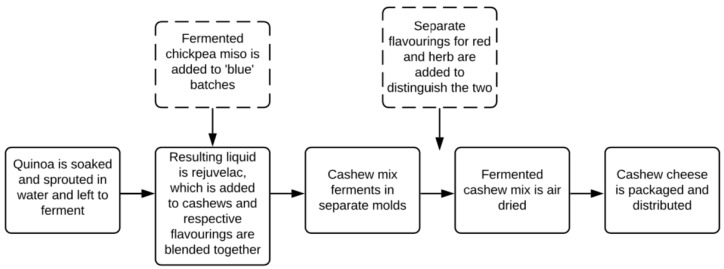
The cashew product fermentation process.

Raw cashews were soaked and blended at room temperature with an inoculated liquid culture termed “rejuvelac.” Rejuvelac was obtained from the retained liquid portion of sprouting grain crop quinoa in water for approximately two days and after reaching pH 3.80-4.30. After adding the rejuvelac and blending, fermentation was allowed to take place for 2–4 days. Due to the mild taste of cashew, several flavour pairings, including herbs and other flavourings, were added to the cashew mix to yield five different flavours: ‘brie’, ’blue’, ’cheddar’, red, and herb (Figure 1), which emulate their dairy equivalents. The herb and red flavourings were only added to the final product, so their initial cashew mixes were identical to each other. The ‘blue’ and ‘cheddar’ varieties included the addition of fermented chickpea miso for additional flavouring (Figure 2).

### 2.2. Product Sampling

Two product batches were followed for sample collection, termed ‘brie’ and ‘blue’. Three samples of a thousand grams were taken from pre-fermentation, post-fermentation, and final product at the production facility (Nuts for Cheese, London, ON, Canada) and were collected from each batch. Eight-hundred grams of each sample were sent for nutritional analysis, five grams were used for pH testing, five grams were used for microbial culturing, five grams were used for allergenicity testing, and one gram was used for microbiota composition analysis. For allergenicity testing, five different varieties (‘cheddar’, ‘blue’, ‘brie’, herb, and red) were followed and fifty gram samples were collected at 0 h, 24 h, 48 h, and 72 h post inoculation. For the red batch, only the 72 h timepoint was collected, because it has the same cashew processing as the herb batch up until 72 h. Five grams were used for allergenicity tests. All samples were stored at 4 °C until needed. 

### 2.3. Nutritional Analysis

Samples of the ‘brie’ product were sent to an accredited food testing laboratory (SGS Canada Inc., Burnaby, BC, Canada) for nutritional analysis by standard methods. The standards and respective protocols used include AFVAN-SLMF-0010 and AOAC 996.06; AFVAN-SLMF-0014 and AOAC 990.03; AFVAN-SLMF-0013 and AOAC 974.29 and AOAC 976.26; AFVAN-SLMF-0018 and AOAC 982.14; AFVAN-SLMF-0022 and AOAC 2011.14; AFVAN-SLMF-0009 and journal of AOAC vol 86, No. 2, 2003 pg 367-373; AFVAN-SLMF-0012 and AOAC 926.08; AFVAN-SLMF-0022 (ICP-MS) and AOAC 926.08 (AOAC International, Rockville, MD, USA). Analyses were conducted for nutritional label content (moisture, fat, protein, carbohydrate, cholesterol, sugar, dietary fibre, iron, calcium, sodium, potassium, ash content, and energy), as well as vitamins B1, B2, B6, B12, and minerals (copper, magnesium, zinc, selenium, manganese, nickel, and phosphorous). All analyses were performed for pre-fermentation, post-fermentation, and final product samples except for the mineral scan, which was performed on the final product sample, only because metals are not degraded or produced by bacteria and yeast [19]. In addition, the pre-fermentation, post-fermentation, and final product sample of ‘brie’ and ‘blue’ batches were tested for pH. Five grams of each sample were mixed with 50 mL of water and then the pH of the mixture was read with a pH probe at 25 °C (Beckman Coulter, UT). The pH of each mixture was recorded four times and the average was calculated. The pH of the original samples accounting for dilution was then calculated.

### 2.4. Culturing of Microorganisms

One gram of each sample was manually homogenized with 10 mL of phosphate-buffered saline (PBS) in a sampling bag (Whirl-Pak, Nasco, ON). The homogenized solutions were then serially diluted 10-fold to 10^−6^ in PBS. All dilutions were then each spread plated onto de Man, Rogosa, and Sharpe (MRS; BD Difco, MD), Rogosa (BD Difco, MD), Sabouraud dextrose agar (SDA; BD Difco, MD), and glucose yeast calcium carbonate agar plates (GYC) for the detection of acetic acid bacterial producers, yeast, and fungi [20]. Plates were aerobically incubated at 37 °C for 3 days.

### 2.5. PCR and 16S rRNA Gene Analysis of Bacterial Isolates and 18S rRNA Gene Analysis of Yeast Isolates

Colonies that appeared morphologically different on agar plates were isolated and their DNA was extracted using the InstaGene matrix (Bio-Rad, CA) for PCR. Suspected bacterial DNA was subjected to PCR using the bacterial ribosomal 16S primers pA (AGAGTTTGATCCTGGCTCAG) and pH (GCGACAAACCACCTACGAG). Suspected yeast DNA was subjected to PCR using the yeast ribosomal 18S primers F-566 (CAGCAGCCGCGGTAATTCC) and R-1200 (CCCGTGTTGAGTCAAATTAAGC). Both bacterial and yeast PCR were carried out using Q5 High-Fidelity DNA Polymerase (New England BioLabs, MA). The bacterial PCR conditions used were an initial denaturation at 95 °C for 4 minutes, followed by 35 cycles of 95 °C for 30 seconds, 55 °C for 30 seconds, and 68 °C for 1 minute. The yeast PCR conditions were an initial denaturation at 94 °C for 4 minutes followed by 40 cycles of 94 °C for 1 minute, 54 °C for 1 minute, and 72 °C for 1 minute. The PCR products were then run on a 1% agarose (w/v) electrophoresis gel for 80 minutes at 80V. The gel was stained with an ethidium bromide wash (0.5 µg/mL) and visualized on a MultiImage Light Cabinet (Alpha Innotech Corporation, CA). The PCR amplicons were then purified with the Invitrogen Purelink PCR Purification Kit (ThermoFisher Scientific, CA). The purified PCR amplicons were analysed with a DeNovix spectrophotometer (model DS-11 (M), DeNovix, DE) for purity and then sent to the London Regional Genomics Centre (Robarts Research Institute, London, ON, Canada) for Sanger sequencing. DNA sequences were compared to sequence databases using the Basic Local Alignment Search Tool (BLAST) for species identification. 

### 2.6. Microbiota Extraction, Sequencing, and Analysis

DNA was extracted using the DNeasy Powersoil Kit (Qiagen, MD). The Beckman BioMek 3000 Laboratory Automation Workstation was set to aliquot 10 µL (2.3 pmol/µL) of 32 primers (16 left and 16 right) with unique barcodes into a 96 well plate. The V4 region of the 16S ribosomal RNA gene was amplified using the primers (5’-3’) ACACTCTTTCCCTACACGACGCTCTTCCGATCTNNNNxxxxxxxxGTGCCAGCMGCCGCGGTAA and (5′-3′) CGGTCTCGGCATTCCTGCTGAACCGCTCTTCCGATCTNNNNxxxxxxxxGGACTACHVGGGTWTCTAAT (where xxxxxxxx is a sample-specific nucleotide barcode, the preceding sequence is a portion of the Illumina adapter sequence for library construction). Then, 2 µL of template DNA and 20 µL of Promega GoTaq Colourless Master Mix (Promega, WI) were added to the well plates. The PCR conditions used were 2 minutes at 95 °C, then 25 cycles of 95 °C for 1 minute, 50 °C for 1 minute, and 72 °C for 1 minute. PCR amplicons were sent to the London Regional Genomics Centre (Robarts Research Institue, London, ON, Canada), where samples were DNA quantified (Quant-it, Life Technologies, ON) and cleaned with QIAquick (Qiagen, MD). DNA was sequenced with the MiSeq Illumina® platform processed with dada2 (version 1.8) and custom R (version 3.4.1) scripts (Dr. Greg Gloor, github.com/ggloor/miseq_bin).

### 2.7. Cashew Allergenicity Testing of Products at Different Stages of Production

For allergen content testing, the BioFront Technologies Cashew ELISA Kit (BioFront Technologies, FL), designed for the sensitive detection of cashew allergens in food products, was adapted for the assay. Preliminary studies using this ELISA assay indicated that samples required the significant dilution of samples in the order of 10^−5^. Other steps of the ELISA were followed as per the manufacturers’ instructions and read using a spectrophotometer microplate reader (BioTek Eon Microplate, VT). A standard curve was created, and sample allergen quantities were calculated against these values.

## 3. Results

### 3.1. Nutritional Analysis

The starting cashew mixtures were both pH 3.0, marginally decreasing to 2.8 in the ‘brie’ final product and 2.9 in the ‘blue’ final product (Table 1). In order to document the possible nutritional changes during nut fermentation, different ‘brie’ timepoint samples (pre-fermentation, post-fermentation, final product) were nutritionally analysed for comparison. The fats, vitamin B12, and moisture decreased when comparing the final product to pre-fermentation samples (Table 1). Conversely, the carbohydrates, energy, sodium, calcium, iron, potassium, vitamin B1, and vitamin B6 increased from pre-fermentation samples. The trans-fat, omega 3 fat, cholesterol, sugars, fibre, vitamin C, and vitamin B12 were not detected at high enough levels to yield a discrete value throughout all three samples. Copper, magnesium, zinc, selenium, manganese, nickel, and phosphorous were only analysed from the final product for quantification and they were present at low levels. 

### 3.2. Viable Microbial Counts

**Figure 3:**
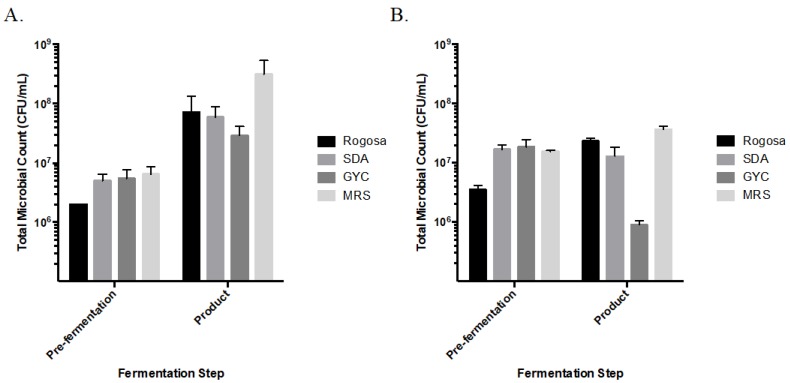
Total microbial colony counts (CFU/mL) of fermented cashew product samples. (**A**) ‘Brie’ and (**B**) ‘blue’ cashew product. Mean and standard deviation are presented. Data are in duplicates.

‘Brie’ samples showed approximately a one log increase of CFU when grown on Rogosa (lactobacilli), SDA (yeast), and GYC (lactic acid bacteria) (Figure 3A). ‘Brie’ samples grown on MRS (semi-selective for lactobacilli-and related bacteria) showed approximately a two log increase (Figure 3A). The highest count of 10^9^ CFU/mL was on MRS media at the product step. The other three media showed increasing counts from 10^7^ to 10^8^ CFU/mL following fermentation. Conversely, the samples from ‘blue’ product showed no log decreases in counts on SDA and MRS, and remained at 10^8^ CFU/mL. The ‘blue’ samples did show a log increase on Rogosa and a two log decrease on GYC (Figure 3B).

### 3.3. Identification of Bacterial Isolates

Bacteria were isolated and identified as *Weissella cibaria*, *Leuconostoc citreum*, and *Lactococcus lactis* (Table 2). The *W*. *cibaria* isolates were identified in both rejuvelac and samples from the ‘blue’ product. *W. cibaria* was found to grow on both MRS and GYC, while *L. citreum* grew on both SDA and GYC. 

### 3.4. Microbiota Characterisation of Fermentation Stages and Products

**Figure 4:**
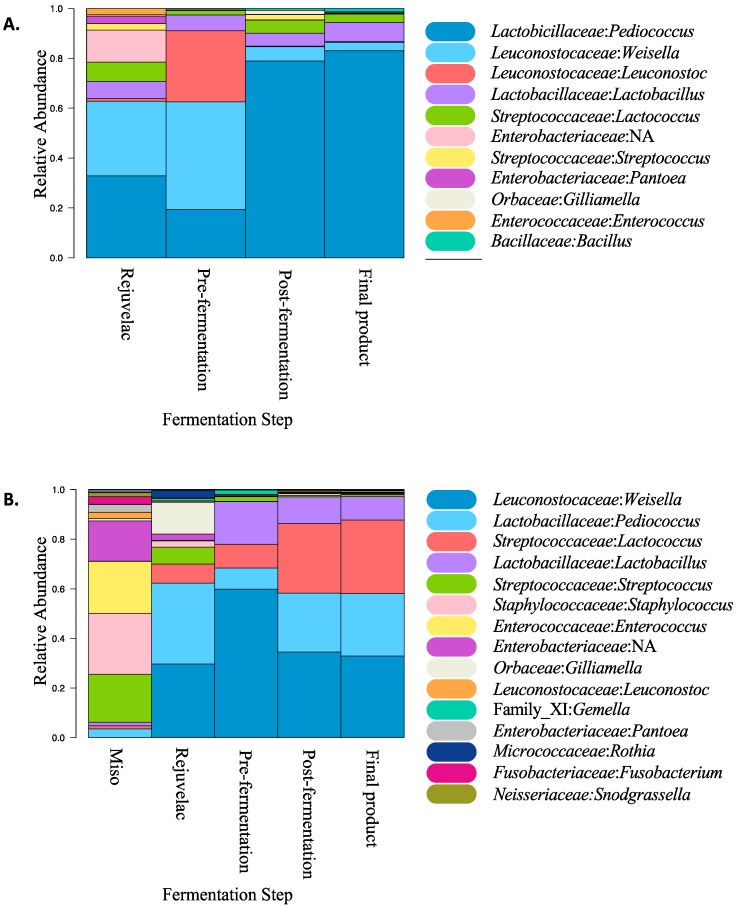
Relative microbiota compositions of fermented cashew products (**A**) ‘brie’ and (**B**) ‘blue’. The V4 region of the 16S rRNA gene was selected for PCR.

The microbiota composition of samples taken from the rejuvelac, miso paste, pre-fermentation, post-fermentation, and final product were examined. Over 17 genera were detected. Comparison of the relative abundance of bacterial composition obtained by 16S rRNA gene sequencing methodologies illustrated that the fermentation of the product alters the microbiota composition, to a few genera dominating the final product (Figure 4). The starting fermented cultures of rejuvelac and miso also influenced the microbial composition of the final product. In the samples from ‘brie’, *Pediococcus* became the most prominent genus in the final product, whereas in the other ‘blue’ samples, the composition was dominated mostly by *Weissella*, *Lactococcus*, and *Pediococcus*. 

### 3.5. Cashew Allergenicity

**Figure 5:**
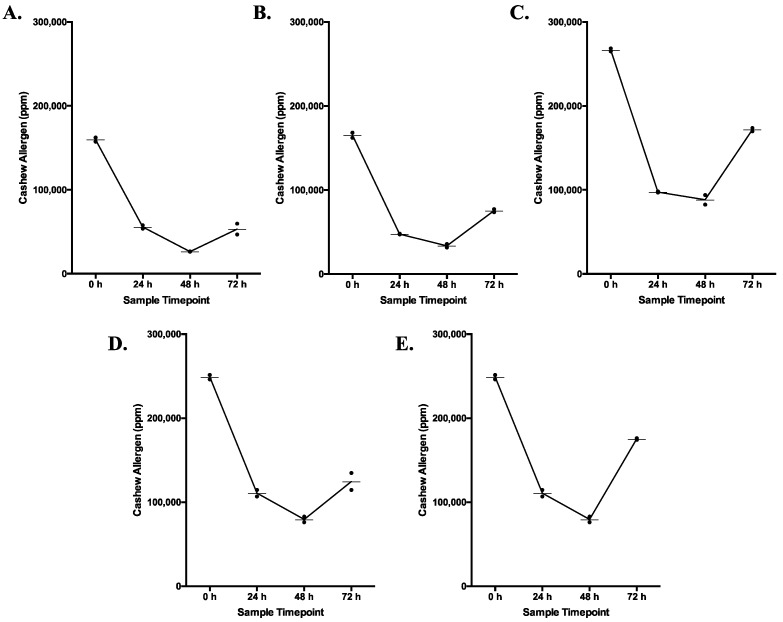
Cashew allergen concentration (ppm) through four timepoints of five fermented cashew products: (**A**) ‘cheddar’, (**B**) ‘blue’, (**C**) ‘brie’, (**D**) herb, and (**E**) red. Each batch was followed from 0 h to 72 h post inoculation, except red which was only collected at 72 h because it has the same fermentation as herb up until 48 h. Samples were diluted x10,000 and run on the BioFront Technologies Cashew ELISA Kit in duplicates. Each point represents one replicate. Horizontal lines represent the mean.

In all five batches that were tested, there was a decrease in cashew allergen content when comparing pre-fermentation (0 h) to the finished product stage (72 h) (Figure 5). Most of the reduced allergy activity occurred within the first 48 hours of fermentation. The ‘brie’ samples started with an allergen concentration of 266,800 ppm and decreased by 35% at 72 h. As for ‘blue’ samples, the allergen concentration started at 165,200 ppm and decreased by 54% after 72 h. Following the same 0 h to 72 h comparison, the ‘cheddar’ started at 159,850 ppm and decreased by 66%, herb started at 248,750 ppm and decreased by 49%, and red started at 248,750 ppm and decreased by 29%.

## 4. Discussion

In this study, a variety of bacterial genera were identified in the production process of fermented cashews. *Weissella* were dominant in rejuvelac and ‘blue’ samples, *Lactococcus* in ‘blue’ samples, and *Leuconostoc* in ‘brie’ samples. As the pH of the fermented cashew mixes ranged from 2.8–3.0, the bacteria were clearly able to tolerate acidic environments and grow on SDA media. Conversely, yeast were not cultured on SDA media perhaps due to low level presence in the rejuvelac to begin with. Certain bacteriocins produced by lactic acid bacteria also have antifungal properties [21].

In the cashew ‘brie’ product, *Pediococcus*, *Weissella* and *Lactobacillus* accounted for nearly 60% of the relative bacterial composition of the final product. These organisms originated from the rejuvelac and they have also previously been isolated from quinoa [22]. Comparing the pre-fermentation, post-fermentation, and final product samples of cashew ‘brie’, these three genera became predominant. *Pediococcus* is commonly found in fermented food, as it plays a role in souring fermented food through the metabolism of carbohydrates into lactic acid [23]. *Weissella* strains are able to metabolize carbohydrates for energy and produce lactic acid and acetic acid through this sugar metabolism [24]. *Lactobacillus* is another genus that is common in fermentation, as it is also capable of producing lactic acid [25]. These three genera each contribute to the maintenance of acidity of the cashew product. 

As for the ‘blue’ product, *Weisella*, *Pediococcus*, and *Lactococcus* cumulatively contributed over 80% of the relative bacterial composition in the final product. *Lactococcus* was present because it is also capable of lactic acid fermentation, thereby contributing to the acidity of the final product, similar to *Weissella* and *Pediococcus* [1].

The rejuvelac used for both ‘brie’ and ‘blue’ products had *Enterobacteriaceae* present at low levels, which may be attributed to contaminated quinoa because *Enterobacteriaceae* have been found to be present in cereal grains and are also common contaminants of food in general [26,27,28]. Although *Enterobacteriaceae* were initially present in the rejuvelac, by the end of cashew fermentation it was no longer detectable. Reduction of *Enterobacteriaceae* may be attributed to the activity of bacteriocins produced by lactic acid bacteria, which are the main bacteria present in both ‘brie’ and ‘blue’ products. Bacteriocins produced by lactic acid bacteria have demonstrated bactericidal and bacteriostatic properties against common food-contaminating *Enterobacteriaceae* genera, such as *Salmonella* and *Listeria* [29,30]. An alternate explanation for the reduction of *Enterobacteriaceae* would be that the cashew mix is more acidic than the starting rejuvelac culture, and some *Enterobacteriaceae* growth is inhibited by acidity [31]. 

Previous studies have demonstrated that many fermented foods are a reliable source for lactic acid bacteria, with counts of 10^5^-10^7^ CFU/mL or g [32]. These include the genera found in this study. The lactic acid preservative effects lower the pH levels past the point of optimal growth for other microorganisms [33]. In both the ‘brie’ and ‘blue’ batches, the pH essentially did not change, further enhancing food safety during cashew fermentation [34].

The bacterial counts stayed within 10^7^–10^9^ CFU/mL or 10^8^–10^10^ CFU/g depending upon the stage of production. The increase in counts after fermentation can be explained by the proliferation of bacteria in the nutrient rich cashew mix. These counts are higher than the levels generally found in fermented foods which usually contain 10^5^–10^7^ CFU/mL or g of lactic acid bacteria [32]. The exception is fermented dairy products with cell counts up to 10^9^ CFU/mL [32]. The high presence of bacteria might imply that the bacteria are metabolizing molecules into by-products that increase the product’s nutrient content. However, this was not noted in this study. Where the basic need for food is no longer caloric intake, the prevention of nutritional diseases and maintenance of health has become a critical part of North American dietary culture [35]. As a result of this, being able to design food beyond just taste, but also to include necessary nutrients and biologically active molecules could be a leverage point for food manufacturers. 

Nutritional analysis revealed an increase then decrease of nutrient levels, which may be attributed to the production and then usage by the microorganisms present. For example, a decrease in fats may be attributed to lipolysis by bacteria. Lipolysis is a natural occurrence in dairy fermentation and it involves the breakdown of triglycerides into fatty acids, which can aid flavour development [36]. Since total fat is the largest component of cashew nuts by weight, contributing approximately 48.3%, there are high levels available for lipolysis in the cashew product [37]. 

Metal analyses were only performed on the final product sample because they can neither be created or destroyed by microorganisms [19]. The levels for copper, magnesium, zinc, selenium, manganese, nickel, and phosphorous were quite low with regards to nutritional requirements. The highest was 2059 ppm of phosphorous or approximately 0.006177 mg per serving. The recommended dietary allowance for adults in Canada for phosphorous is 700 mg per day [38]. 

A potentially important finding was that cashew fermentation yielded a decrease in cashew allergen in all varieties of product that were tested. Most of the decrease appeared in the first 48 hours. From 48 h to 72 h, a slight increase was observed; however, this may be due to the evaporation of moisture from the product as they are dried from 44.0% to 43.4%, which proportionally increases the cashew content. The decrease in cashew allergen is likely from proteolytic cleavage by a bioactive molecule produced during fermentation, as this is the mechanism shown to decrease peanut allergenicity [39,40]. Finding methods to overcome nut allergies is a field of increasing interest as the prevalence of nut allergies is a major contributor to anaphylaxis in children [18]. Immunotherapy is a major avenue that is pursued when attempting to mitigate nut allergies, but it has its drawbacks with limited results [16]. An alternative to immunotherapy is decreasing nut allergens in food so that an allergic reaction is never reached. Methods to remove cashew allergens such as with p-aminobenzamidine and magnetic 6% cross-linked agarose beads have yielded success in decreasing some cashew allergens, but not all [14]. With peanuts, biological methods to decrease allergenicity have been investigated and enzymatic cleavage of peanut allergens, such as seed storage proteins, Ara h1, Ara h 2, and Ara h 3, have been shown to decrease along with its IgE binding reactivity (17–19). Cashews have similar proteins to peanuts as their major allergens and therefore may be subject to alike enzymatic cleavage to decrease allergenicity. The findings suggest that bacteria degraded one or more of the three major cashew allergen proteins, Ana o 1, Ana o 2, and Ana o 3 [41,42,43,44], which are associated with higher rates of anaphylaxis compared to peanuts [45]. Currently, there is no enzymatic treatment of cashew allergens. The microbiota sequencing identified lactic acid bacteria as predominant throughout the cashew product samples, and it appears that their proteolytic activity was involved in the reduction in allergy activity [46]. Although the cashew allergen load decreased during fermentation, it did not decrease past the threshold of eliciting cashew allergies of approximately 60 ppm per cashew ‘cheese’ serving size [47]. Despite this, decrease in cashew allergen via fermentation is a method that could be useful in decreasing allergens.

In summary, the study cashew products retained their nutritional composition after fermentation, and the starter rejuvelac culture was important in generating the end-product. The reduction in cashew allergens with fermentation provided an additional benefit for consumers. 

## Figures and Tables

**Table 1:** Nutritional compounds and pH analyzed from fermented cashew cheese ‘brie’ samples at three time points (pre-fermentation, post-fermentation, final product).

Compound	Pre-Fermentation	Post-Fermentation	Final Product	Unit
saturated fat	18.6	19.7	18.2	%
monounsaturated fat	11.3	12.4	10.9	%
polyunsaturated fat	3	3.3	2.9	%
trans fat	<0.1	<0.1	<0.1	%
total fat	35.1	37.6	33.9	%
omega 3 fat	<0.1	<0.1	<0.1	%
omega 9 fat	11.2	12.4	10.8	%
omega 6 fatty acids	3	3.3	2.9	%
calories from fat	316	338	305	cal/100 g
crude protein content	8.3	8.1	8.2	%
crude ash content	2.5	1.5	1.4	%
carbohydrates	10.2	8.9	13.1	%
energy	389	406	390	cal/100 g
energy	1629	1700	1632	kJ/100 g
cholesterol	<2	<2	<2	mg/100 g
fructose	<0.2	<0.2	<0.2	g/100 g
glucose	<0.2	<0.2	<0.2	g/100 g
sucrose	<0.2	<0.2	<0.2	g/100 g
maltose	<0.2	<0.2	<0.2	g/100 g
lactose	<0.2	<0.2	<0.2	g/100 g
total sugar	<0.2	<0.2	<0.2	g/100 g
total fibre	<0.4	<0.4	<0.4	%
sodium	610	591	626	mg/100 g
calcium	30	31	41	mg/100 g
iron	1.6	2.6	1.7	mg/100 g
potassium	171	167	176	mg/100 g
vit C	<2	<2	<2	mg/100 g
vit B1, HCl	0.00118	0.00107	0.00123	%
vit B2	0.00097	0.00044	0.00078	%
vit B6	0.00098	0.00108	0.00112	%
vit B12	<0.1	<0.1	<0.1	ppm
moisture	44	43.9	43.4	%
copper	-	-	8.6	ppm
magnesium	-	-	732.9	ppm
zinc	-	-	17	ppm
selenium	-	-	<0.1	ppm
manganese	-	-	6.7	ppm
nickel	-	-	0.8	ppm
phosphorous	-	-	2059	ppm
pH of ‘brie’	3.0	2.9	2.8	-
pH of ‘blue’	3.0	2.9	2.9	-

% unit represents % of sample analyzed; pH values represent the mean of 4 pH readings, each taken at 25 °C.

**Table 2:** Comparison of species sequenced from cultured isolates vs. genera sequenced from microbiota analysis.

Sample	Species and Media	Genera
Rejuvelac	*Weisella cibaria* (MRS)	*Weisella*
	*Weisella cibaria* (GYC)	*Weisella*
‘Brie’	*Leuconostoc citreum* (SDA)	*Leuconostoc*
	*Leuconostoc citreum* (GYC)	*Leuconostoc*
‘Blue’	*Lactococcus lactis* (MRS)	*Lactococcus*
	*Weisella cibaria* (MRS)	*Weisella*
